# Phosphorylation of apoptosis repressor with caspase recruitment domain by protein kinase CK2 contributes to chemotherapy resistance by inhibiting doxorubicin induced apoptosis

**DOI:** 10.18632/oncotarget.4392

**Published:** 2015-06-27

**Authors:** Jianxun Wang, Chang Feng, Yuqi He, Wei Ding, Jianqiu Sheng, Muhammad Arshad, Xiaojie Zhang, Peifeng Li

**Affiliations:** ^1^ Institute for Translational Medicine, Medical College of Qingdao University, Qingdao, 266021, China; ^2^ National Key Laboratory of Biomembrane and Membrane Biotechnology, Institute of Zoology, Chinese Academy of Sciences, Beijing, 100101, China; ^3^ Department of Gastroenterology, Beijing Military General Hospital, Beijing, 100700, China; ^4^ Affiliated Hospital, Medical College of Qingdao University, Qingdao, 266003, China

**Keywords:** chemotherapy resistance, ARC, CK2, apoptosis, doxorubicin

## Abstract

The development of cancer resistance to chemotherapy is the major obstacle to cancer therapy. Here, we identified that the phosphorylation of apoptosis repressor with caspase recruitment domain (ARC) at threonine 149 was essential to inhibit doxorubicin (DOX) induced apoptosis and mitochondrial fission in cancer cells. Our further study showed that casein kinase II (CK2) inhibitors could decrease the phosphorylation levels of ARC and make cancer cells sensitive to undergoing apoptosis. Furthermore, CK2α and CK2α', catalytic subunits of CK2, were observed to translocate into nuclear in cancer cells with the treatment of DOX. Finally, the synergistically therapeutic effect by combining DOX and CK2 inhibitor was confirmed in tumor xenograft model. Taken together, our results revealed that CK2-mediated phosphorylation of ARC contributed to chemotherapy resistance by inhibiting DOX induced apoptosis and combining DOX with CK2 inhibitor could induce apoptosis of cancer cells synergistically by down-regulating the phosphorylation of ARC. Therefore, development of new therapeutic strategies based on ARC and CK2, is promising for overcoming cancer resistance to chemotherapy.

## INTRODUCTION

The development of cancer resistance to chemotherapy during treatment is a major obstacle to cancer therapy [1]. The ultimate goal of chemotherapies is to induce tumor cell death, while the suppression of apoptosis contributes to drug resistance in cancer cells [2, 3]. Doxorubicin (DOX) is widely used in cancer treatments. However, many cancers have developed resistance to this chemotherapy drug which became major barriers to its clinical application [4, 5]. Considering that apoptosis associates with the development of anti-drug characteristics, it matters a lot to reveal the underlying mechanisms of apoptosis triggered by DOX in cancer cells and then provoke the apoptosis inducing effect to improve the efficiency of this anticancer drug.

Apoptosis repressor with caspase recruitment domain (ARC) is an anti-apoptosis protein initially discovered in the skeletal muscle and the heart [6]. It is reported that ARC is highly expressed in many malignant tumors [7, 8]. Our previous work has proved that highly expressed ARC contributed to chemotherapy resistance in cancer cells by targeting the mitochondrial fission machinery [9]. We also observed that ARC expression was down-regulated in cancer cells following DOX treatment. Phosphorylation and dephosphorylation play a critical role in ARC activity regulation [10, 11]. Casein kinase II (CK2) can phosphorylate ARC at threonine-149 (T149) to protect cardiomyocytes against oxidative stress-induced apoptosis [10]. Meanwhile it's unknown whether ARC is phosphorylated in cancer cells and whether T149 phosphorylation is essential for cancer resistance to chemotherapy. The involvement of phosphorylated ARC in chemoresistance needs to be further investigated.

There are arguments about the subcellular localization of ARC in cancer cells [12, 13]. It's reported that ARC is predominantly distributed in the nuclei of some human cancer cell lines [13]. However, in other cancer cell lines such as human melanoma cell line ARC is predominantly distributed in cytoplasm [12] and our previous study found that ARC was localized in cytoplasm in human gastric cancer SGC-7901 cells and human cervical carcinoma HeLa cells [9]. Another study shows interesting results that ARC locates only to cytoplasm in differentiated or poorly differentiated colon adenocarcinoma, meanwhile ARC locates to both cytoplasm and nucleus in moderately differentiated colon adenocarcinoma [7]. All these indicate the subcellular localization of ARC is cell type dependent. It has been also shown that the localization of ARC is regulated by phosphorylation. CK2 phosphorylates ARC at T149 enabling it to translocate to mitochondria in cardiomyocytes, whereas the non-phosphorylated ARC is located in the cytoplasm [10, 14]. The cytolocalization and the functions of phosphorylated ARC in cancer cells need to be further clarified.

CK2 is a constitutively active serine/threonine kinase ubiquitously localized in nucleus and cytoplasm. Nearly all cancers that have been examined show increased CK2 expression [15]. The CK2 holoenzyme is composed of two catalytic subunits (CK2α and/or CK2α') and two regulatory CK2β subunits while the catalytic subunits can catalyze hundreds substrates and participates in complex cancer regulation networks [16]. It has long been known that CK2 involved in cell growth, proliferation and apoptosis. The specific inhibitor 4, 5, 6, 7-tetrabromobenzotriazole (TBB) of CK2 can make prostate cancer cells more sensitized to apoptosis and overexpression of CK2 can blocked the apoptosis pathway [17, 18]. As CK2 can phosphorylate ARC in cardiomyocytes to prevent apoptosis [10], it is necessary to investigate whether CK2 can target ARC in cancer cells to boost the tumorigenesis and induce the chemotherapy resistance.

The present study was designed to elucidate whether phosphorylated ARC was contributed to chemotherapy resistance in cancer and investigate the potential molecular mechanism. Our results showed that phosphorylated ARC localized to mitochondria and played a major part in chemotherapy resistance in cancer cells. Moreover, CK2 could phosphorylate ARC directly and CK2 inhibitor made cancer cells sensitive to undergoing apoptosis. Furthermore, CK2α and CK2α' translocated into nuclear in cancer cells treated by DOX. The therapy efficacy was confirmed in xenograft tumor model by combining DOX and CK2 inhibitor. Our results identify a novel molecular mechanism for chemotherapy resistance involving phosphorylation of ARC by CK2 and provide a valuable insight into cancer therapy.

## RESULTS

### ARC is phosphorylated and localizes to mitochondria in cancer cells

**Figure 1:**
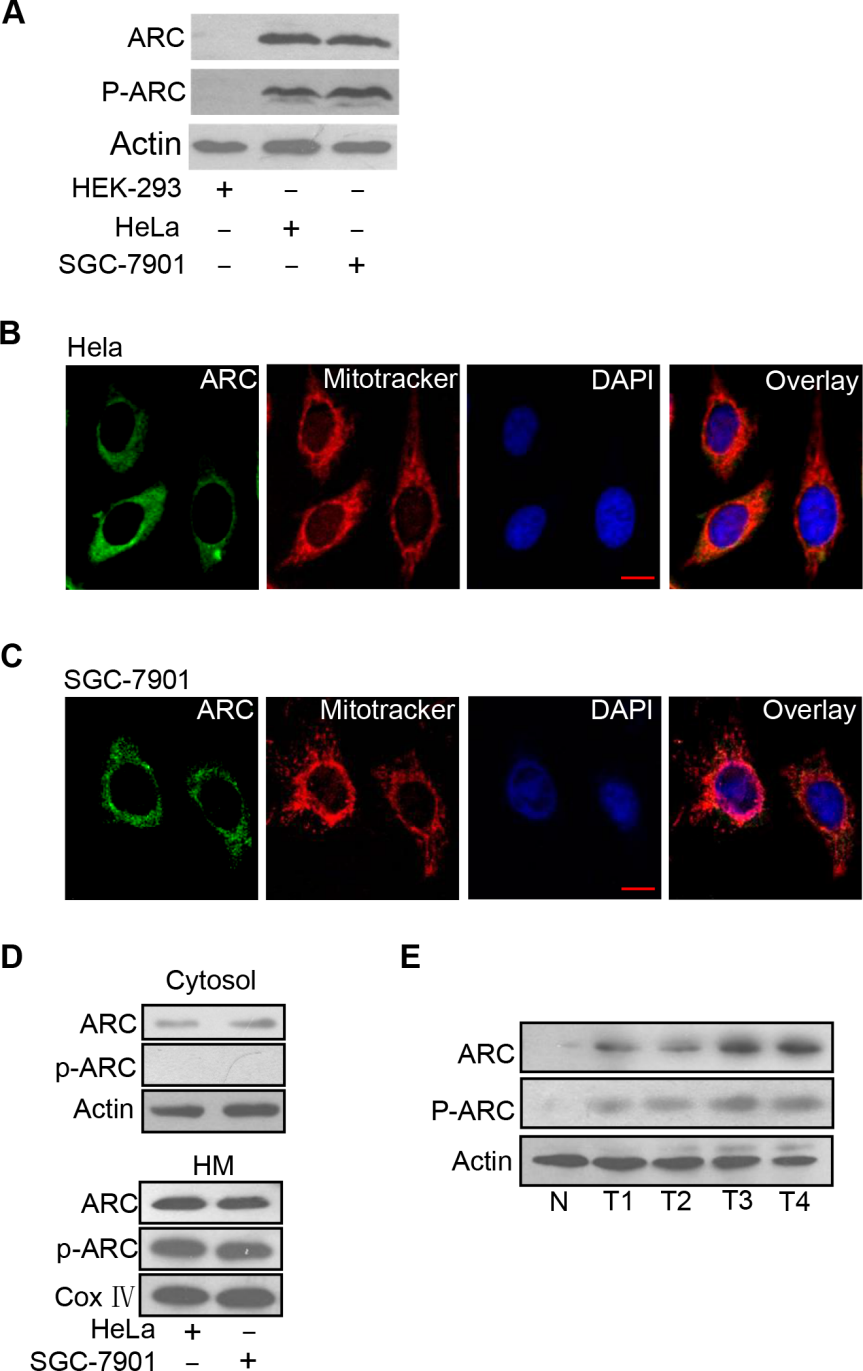
ARC is phosphorylated and localizes to mitochondria in cancer cells **A.** Analysis of total ARC and phosphorylated ARC (p-ARC) expression levels in cancer cells including HeLa and SGC-7901 cells. HEK-293 cells served as a negative control. ARC and p-ARC were detected by immunoblot. A representative result of three independent experiments is shown. **B.** Distribution of ARC was detected in HeLa cells by immunofluorescence. HeLa cells labeled with MitoTracker (red), stained with anti-ARC antibody, and monitored by FITC-labeled secondary antibody (green), and DAPI (blue). **C.** Distribution of ARC was detected by immunofluorescence in SGC-7901 cells which were analyzed as described for B. **D.** HeLa and SGC-7901 cells were harvested for the detection of ARC and p-ARC in the cytosol (top) and mitochondria-enriched HM (bottom). A representative blot of three independent experiments is shown. **E.** Immunoblot of ARC and p-ARC proteins in clinical samples with one normal gastric tissue (N) and four gastric tumor tissues (T).

Recent studies have shown that ARC contributes to chemotherapy resistance in cancer cells [9, 19]. However, the cytolocalization and the functions of phosphorylated ARC in cancer cells remain largely unknown. Firstly, we detected whether ARC is phosphorylated and whether it locates to mitochondrion to perform its function in cancer cell lines. Phosphorylated ARC could be detectable in cancer cells including HeLa and SGC-7901 but not in the normal HEK-293 cells (Figure 1A). ARC localization was demonstrated by immunofluorescence and we found that it was distributed throughout the perinuclear region. It is worthwhile to note that ARC distribution pattern coincided very closely with that of the mitoTracker in HeLa cells (Figure 1B). A similar result was observed in SGC-7901 cells (Figure 1C). The distribution of ARC and phosphorylated ARC in subcellular fractions of mitochondria-enriched heavy membranes (HM) and cytosol was further detected. We discovered the phosphorylated ARC only in HM but not in cytosol (Figure 1D). Significantly expressed phosphorylated ARC was also observed in gastric cancer tissues compared with normal gastric tissues (Figure 1E). These results suggest that ARC is phosphorylated and localizes to mitochondria in cancer cells and clinical cancer tissues.

### ARC requires to be phosphorylated to inhibit DOX induced apoptosis and mitochondria fission

**Figure 2:**
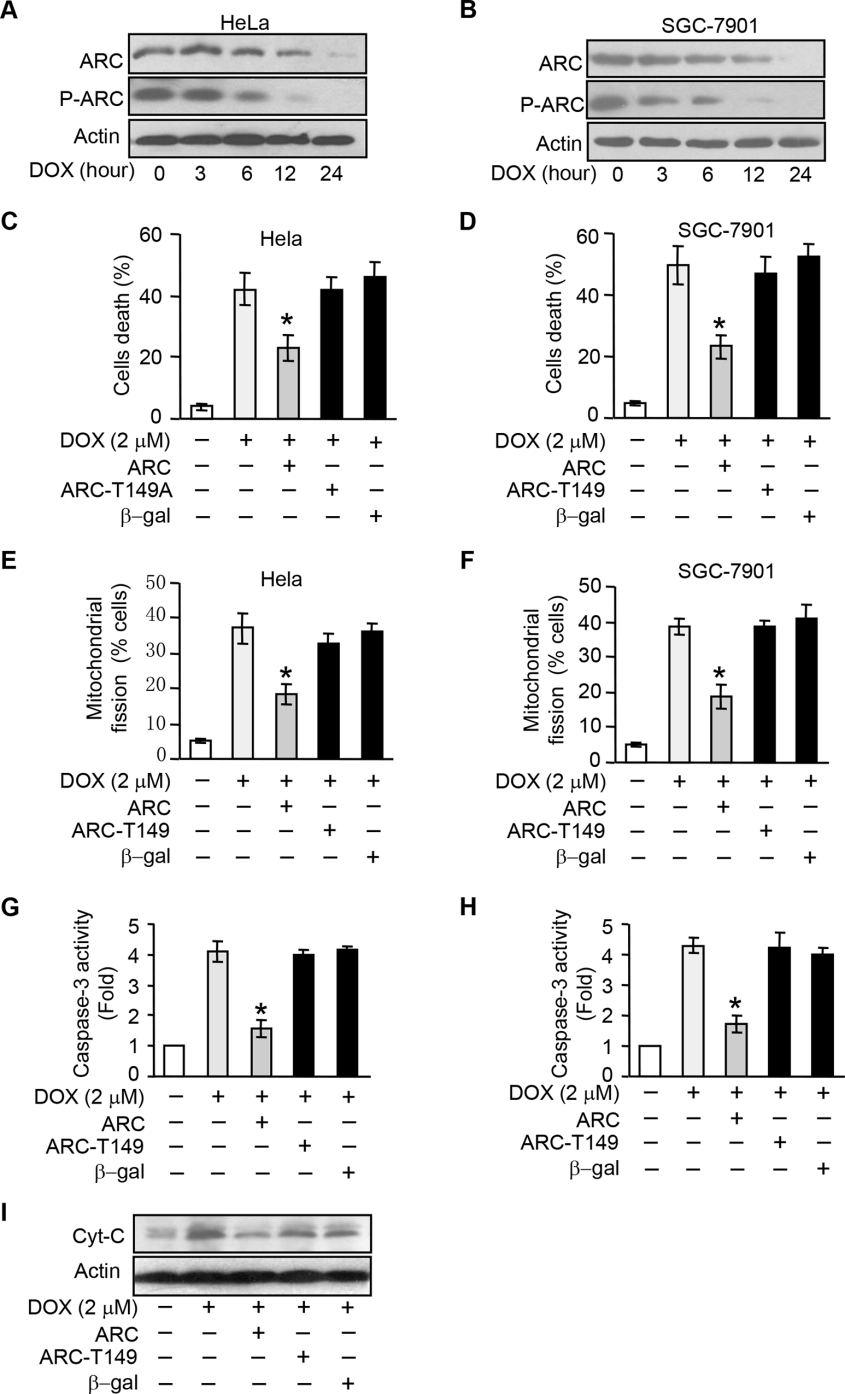
ARC requires to be phosphorylated to inhibit DOX induced apoptosis and mitochondria fission **A–B.** ARC and p-ARC levels were detected in HeLa (A) and SGC-7901 (B) cells treated with DOX (2 μM) at the indicated time. **C–D.** wtARC inhibited DOX-induced cell death. HeLa (C) and SGC-7901 (D) cells were infected with adenovirus ARC, ARC T149A mutant adenovirus (ARC T149A) or adenovirus β-gal. 24 hours after infection they were treated with DOX (2 μM) for 36 hours. Cell death was assessed by trypan blue exclusion. *p < 0.05 vs DOX alone. **E–F.** wtARC but not ARCT149A prevented DOX-induced mitochondrial fission. HeLa (E) or SGC-7901 (F) was infected with adenovirus ARC or ARC T149A. 24 hours after infection they were treated with DOX (2 μM). 12 hours after treatment, mitochondrial fission was detected. *p < 0.05 vs DOX alone. **G–H.** Caspase-3 activities were detection in HeLa (G) or SGC-7901 (H) cells which were treated as in C and D. *p < 0.05 vs DOX alone. **I.** Analysis of cytochrome C (Cyt-C) release in HeLa cells treated as described in C. Data are expressed as the mean ± SD of 3 independent experiments.

To study the role of phosphorylated ARC in DOX resistance, we treated cancer cells with DOX. The expression levels of total ARC and phosphorylated ARC were down-regulated after DOX treatment in a time-dependent manner in HeLa (Figure 2A) and SGC-7901 cells (Figure 2B), and what was noteworthy was that phosphorylated ARC expression levels decreased more sharply than total ARC levels. These results indicated that the decrease of phosphorylated ARC levels was not totally by the results of the decrease of total ARC protein levels, as the phosphorylated ARC levels decreased more obviously than total ARC protein levels. And these results suggested that the phosphorylation of ARC may be inhibited by DOX.

T149 is the functional phosphorylation site of ARC [10]. ARC T149 phosphorylation is required for its relocalization to mitochondria where ARC plays the anti-apoptosis function [10, 11, 20]. To elucidate whether ARC depends on phosphorylation at T149 to resist the DOX induced-apoptosis, we then compared the effect of phosphorylatable wild type ARC (wtARC) and nonphosphorylatable T149 mutant ARC on DOX induced apoptosis. T149 mutant ARC refers to the phosphorylation site threonine149 is mutated to alanine (ARC T149A) and alanine mutation led to the loss of phosphorylation capability of ARC. HeLa and SGC-7901 cells were infected with adenoviral ARC and ARC T149A. Enforced expression of ARC and phosphorylated ARC was confirmed by immunoblot (Supplementary Figure 1A and 1B). DOX-induced cell death could be attenuated by wtARC but not ARC T149A in HeLa and SGC-7901 cells (Figure 2C and 2D). While mitochondrial fission is related to the initiation of apoptosis and phosphorylated ARC locates to mitochondrion, enforced expression of ARC T149A could not inhibit mitochondrial fission as effectively as wtARC in cancer cells (Figure 2E and 2F). To further confirm that is apoptotic cell death, caspase-3 activity assay was performed. The results showed that administration of wtARC but not ARC T149A could inhibit the caspase-3 activity induced by DOX in HeLa and SGC-7901 cells (Figure 2G and 2H). Meanwhile wtARC could attenuate the release of cytochrome C upon DOX treatment in HeLa cells (Figure 2I). We also detected the cellular localization of ARC in HeLa cells infected with wtARC or ARC T149A adenovirus, the results suggested that wtARC but not ARC T149A could relocate to mitochondria (Supplementary Figure 2). Taken together, these results indicate that ARC requires to be phosphorylated at T149 to inhibit DOX induced apoptosis and maintain the mitochondrial morphology in cancer cells.

### Inhibition endogenous ARC phosphorylation by CK2 inhibitor sensitizes cells to undergoing apoptosis

**Figure 3:**
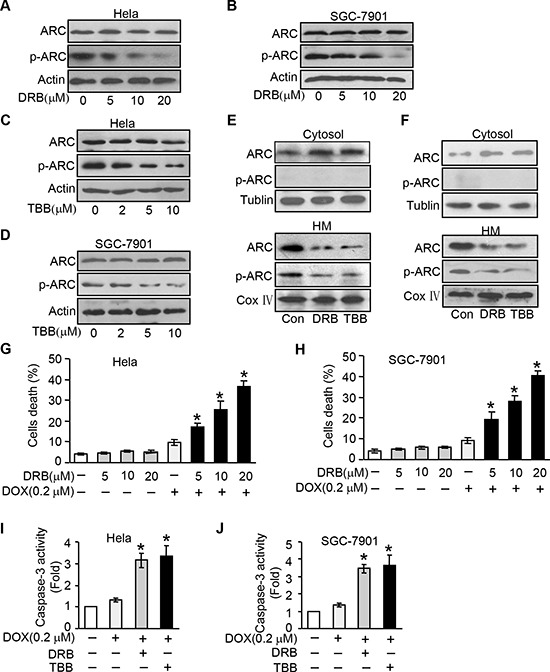
Inhibition endogenous ARC phosphorylation by CK2 inhibitor sensitizes cells to undergoing apoptosis **A–B.** CK2 inhibitor DRB was able to inhibit endogenous ARC phosphorylation. HeLa (A) and SGC-7901 (B) were treated with DRB as indicated concentration for 36 hours, total ARC and p-ARC were detected by immunoblotting. **C–D.** CK2 inhibitor TBB was able to inhibit endogenous ARC phosphorylation in HeLa (C) and SGC-7901 (D). **E–F.** CK2 inhibitor prevents ARC accumulations in mitochondria. HeLa cells (E) and SGC-7901 cells (F) were treated with DRB or TBB for 36 hours. Cells were harvested for the immunoblot analysis of ARC and p-ARC in the cytosol and mitochondria-enriched HM. **G–H.** DRB sensitizes DOX to induce cell death. HeLa (G) and SGC-7901 (H) were administrated with indicated concentration of DRB for 36 hours and then treated with DOX (0.2 μM). Cell death was analyzed 36 hours after treatment. *p < 0.05 vs DOX alone. **I–J.** caspase-3 activity was boosted by combination of CK2 inhibitor and DOX at low dose of 0.2 μmol/L in HeLa (I) or SGC-7901cells (J) *p < 0.05 vs DOX alone. Data are expressed as the mean ± SD of 3 independent experiments.

To better understand the phosphorylation is required for ARC to exert its anti-apoptotic function and exploring the potential mechanism by which ARC be phosphorylated, we detected whether inhibit endogenous ARC phosphorylation can control cancer cell apoptosis. Since it has been shown that CK2 can phosphorylate ARC at Thr149, in order to inhibit the phosphorylation of endogenous ARC we employed CK2 inhibitors 5, 6-dichloro-1-β-D-ribofuranosylbenzimidazole (DRB) and TBB, both of them can specifically inhibit the activity of CK2. We verified that DRB could induce a decrease of the phosphorylated form of ARC in a dose-dependent manner in HeLa and SGC-7901 cells (Figure 3A and 3B). TBB strengthened this statement with similar results (Figure 3C and 3D). Protein kinases such as CK2, p38 mitogen-activated protein kinases (p38), c-Jun N-terminal kinases (JNK) and extracellular signal-regulated kinases (ERK) play an essential role in cancer development and are activated in tumor cells [21, 22]. To further explore whether other kinases can phosphorylate ARC at threonine 149, we detected the phosphorylated ARC protein levels in HeLa cells treated with p38, JNK or ERK inhibitors respectively. The results suggested that the phosphorylated ARC levels were not affected by these kinase inhibitors (Supplementary Figure 3). When CK2 was inhibited, the phosphorylated ARC protein levels demonstrated a decrease in the HM fractions contrasted to the unphosphorylated ARC increased in the cytosolic fractions in HeLa cells (Figure 3E). And we reproduced the same results in SGC-7901 cells (Figure 3F).

Then we attempted to investigate the influence of CK2 inhibitor on cell susceptibility to chemotherapy. Following low-dose DOX (0.2 μM) treatment in cancer cells, a limited amount of cells undergoing death was observed. Whereas when we administrated with DRB or TBB, the death cells were significantly increased in response to the same dose of DOX (Figure 3G and 3H, and Supplementary Figure 4A and 4B). We also found this combination could synergistically boost caspase-3 activity (Figure 3I and 3J). Collectively, our data indicate that inhibition endogenous ARC phosphorylation by CK2 inhibitor make cancer cells more sensitive to DOX.

### CK2 regulates apoptosis through targeting ARC

**Figure 4:**
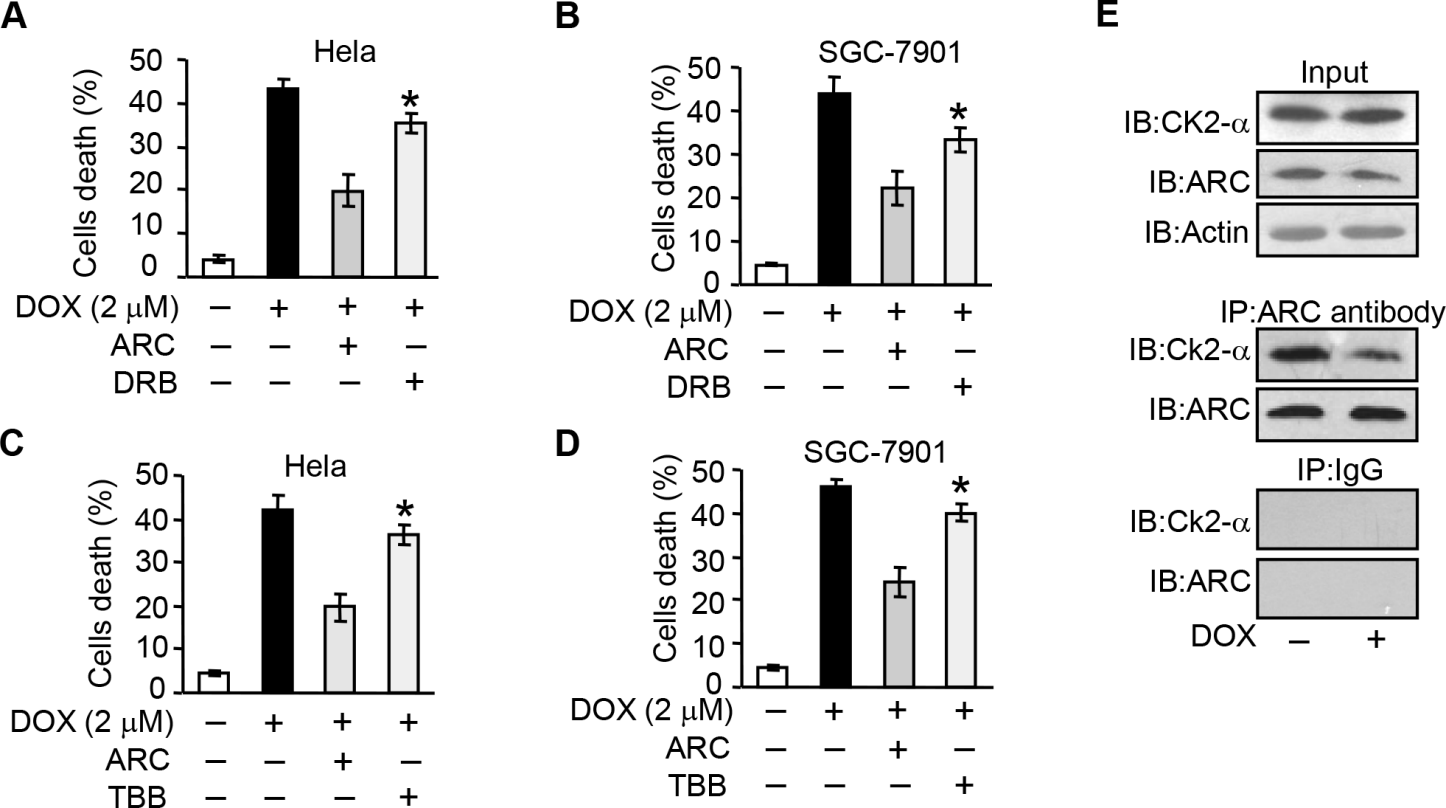
CK2 regulates apoptosis through targeting ARC **A–D.** ARC-attenuated DOX induced cells death is abolished by CK2 inhibitor. HeLa (A and C) or SGC-7901 (B and D) cells were infected with adenovirus ARC, and treated with DRB (A and B) or TBB (C and D) for 36 hours. Cells were exposed to DOX (2 μM). Cell death was analyzed by trypan blue exclusion. *p < 0.05 vs DOX +ARC. **E.** The binding of ARC to endogenous CK2α is reduced in Hela cells exposed to DOX. HeLa cells were treated with DOX (2 μM) for 6 hours and the association between ARC and CK2α was analyzed by immunoprecipitation (IP) followed by immunoblot (IB). Data are expressed as the mean ± SD of 3 independent experiments.

As CK2 is able to phosphorylate ARC, we wondered whether ARC regulated DOX sensitivity in cancer cells to undergoing apoptosis depending on CK2. Enforced expression of ARC inhibited DOX-induced mitochondrial fission and cell death. HeLa and SGC-7901 cells administrated with CK2 inhibitor DRB could abolish the inhibitory effect on cell death (Figure 4A, 4B) in the presence of exogenous ARC. However, DRB itself at the doses used in this study could not induce apoptosis. A similar result was obtained in cancer cells administrated with TBB (Figure 4C, 4D).

To elucidate the direct relationship between CK2 and ARC, we performed immunoprecipitation experiment to detect the association between CK2 catalytic subunits CK2α and ARC. The results revealed that the binding of CK2α and ARC sharply reduced upon DOX treatment (Figure 4E). These data suggest that CK2 phosphorylates ARC directly and contributes to the antagonism of cancer cells to chemotherapy.

### CK2α translates to nuclear in cancer cells exposed to DOX

**Figure 5:**
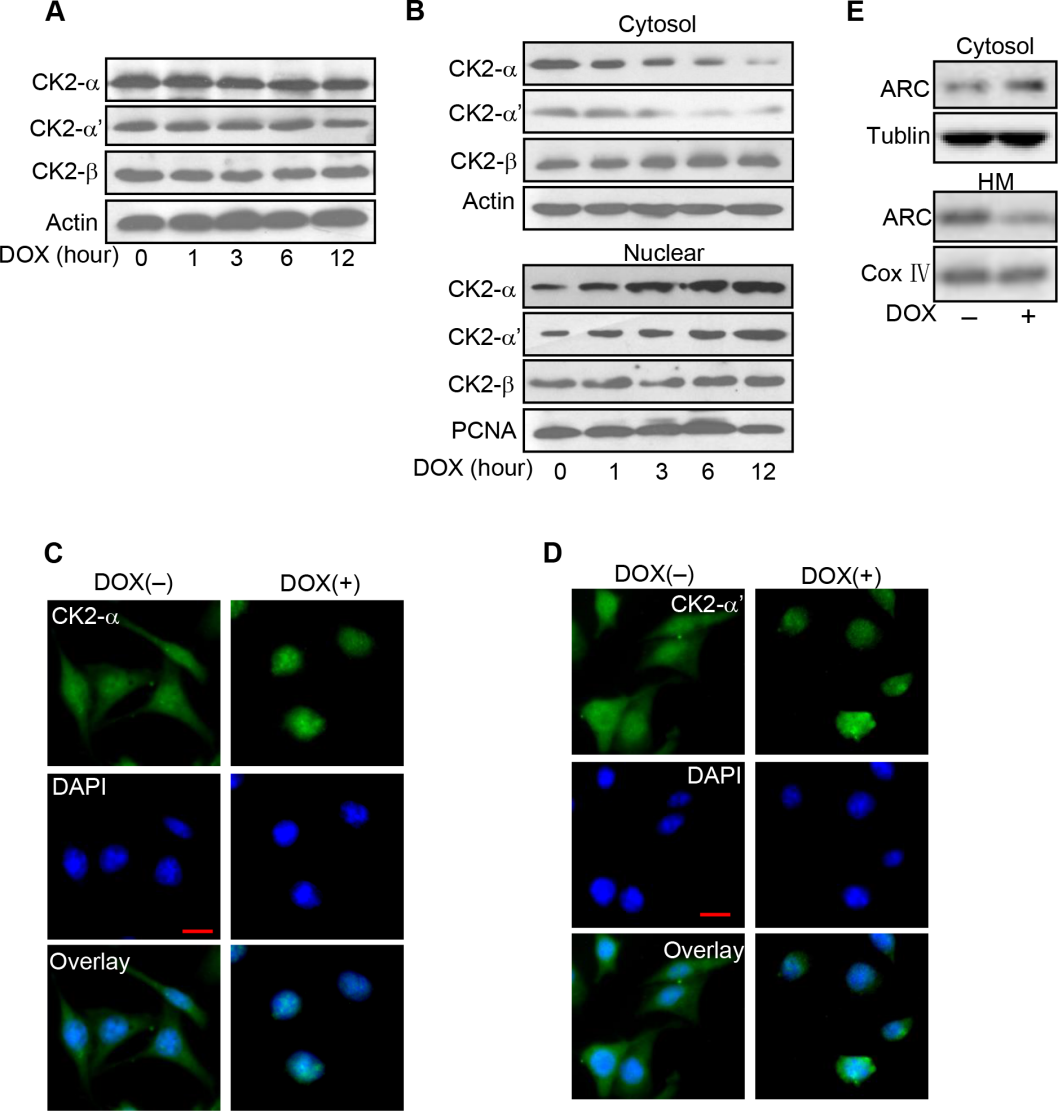
CK2α translates to nuclear in cancer cells exposed to DOX **A.** Analysis of CK2 subunits protein levels in HeLa cells treated with DOX (2 μM) as the indicated time. CK2α, CK2α' and CK2β were detected by immunoblot. **B.** DOX induces CK2α and CK2α' accumulated in nuclear from cytosol. HeLa cells were treated with 2 μM DOX and harvested at the indicated time after treatment for immunoblot analysis of CK2 subunits in the cytosol and nuclear. **C–D.** Distributions of CK2α and CK2α' were detected by immunofluorescence. HeLa cells were labeled with DAPI (blue), stained with anti-CK2α (C) or anti-CK2α' (D) antibody and then monitored by FITC-labeled secondary antibody (green) and DAPI (blue). Bar = 20 μm. **E.** The distribution of ARC in mitochondrial is reduced in HeLa cells exposed to DOX. HeLa cells were treated with DOX (2 μM) for 6 hours and harvested for the immunoblot analysis of ARC in the cytosol and mitochondria-enriched HM. A representative result of three independent experiments is shown.

The prior results showed phosphorylated ARC decreased more sharply than total ARC levels and phosphorylation of ARC was inhibited by DOX. Thus, we attempted to figure out whether the decrease of phosphorylated ARC related to CK2 expression level or activity change in cancer cells treated with DOX. Firstly, we detected the expression levels of CK2 subunits in HeLa cells treated with DOX. As shown in Figure 5A, the protein levels of CK2α, CK2α' and CK2β changed indistinctively with DOX treatment. It's reported that the control of the nucleocytoplasmic distribution of CK2 subunits also play an important role in regulatory mechanism for CK2 activity [23]. And then we detected the subcellular locations of CK2 subunits upon DOX treatment. The levels of CK2α and CK2α' in cytosol were decreased upon the DOX treatment in HeLa cells and their levels in nuclear were increased in the meantime, nevertheless levels of CK2β in cytosol and nuclear both remained constant (Figure 5B). Immunofluorescence was used to further detect the localizations of CK2α and CK2α' in HeLa cells treated with DOX. In the control untreated cells, CK2α was distributed throughout the cellular. Upon DOX treatment, CK2α accumulated into the nuclear (Figure 5C). CK2α' showed the similar migration pattern (Figure 5D). Corresponding to the translocation of CK2 to nuclear, the protein level of ARC was decreased in mitochondrial in HeLa cells treated with DOX (Figure 5E). Thus, it seems that catalytic subunits of CK2 translate to nuclear in cancer cells exposed to DOX could further reduce the activity of CK2 in cytoplasm. While nonphosphorylated ARC is predominantly localized to cytoplasm which means that CK2 catalytic subunits translocate to nuclear and cytosolic CK2 activity decrease might contribute to abolish the phosphorylation of ARC in cytoplasm to make cancer cells more sensitive to DOX.

### DOX combines with CK2 inhibitor enhance chemotherapeutic effect *in vivo*

**Figure 6:**
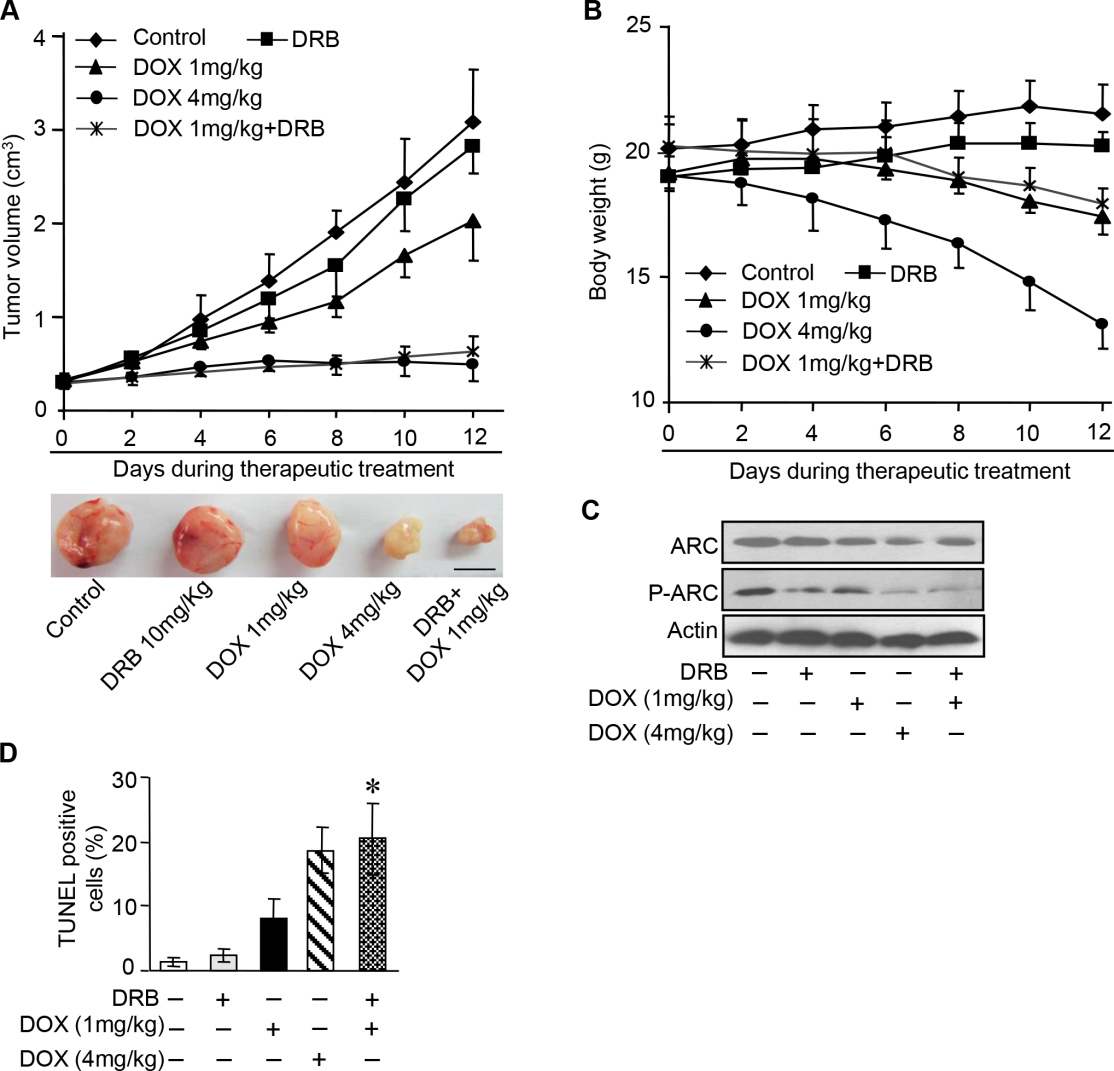
DOX combines with CK2 inhibitor enhance chemotherapeutic effect *in vivo* **A.** A total of 1 × 10^7^ HeLa cells were injected subcutaneously into BALB/c nude mice. When tumors reached 250–300 mm^3^, therapeutic treatment as indicated was given every other day. Tumor volumes were monitored during 2-week therapy (*n* = 6 each group) (top). At the end of this experiment, tumors were dissected and photographed. Representative images of tumors were shown (bottom), scale bar = 1 cm. **B.** Body weight of the tumor bearing mice described in A was measured during two-week therapeutic treatment (*n* = 6 each group). Error bars represent SD. **C.** Immunoblot analysis as indicated in the xenograft tumors described in A. A representative result of three independent experiments is shown. **D.** Apoptosis detected in sections of xenograft tumors described in A by TUNEL assay, *n* = 6 each group. *P < 0.05 compared with DOX (1 mg/kg) alone. Error bars represent S.D.

Having demonstrated CK2 inhibitor can efficiently attenuate the phosphorylation of endogenous ARC and significantly increased the susceptibility of cancer cells to DOX *in vitro*, we further investigated CK2 inhibitor therapeutic potential in mouse xenograft model. HeLa cells were subcutaneously inoculated into nude mice. When the tumor volumes reached 250–300 mm^3^, intraperitoneal delivery of DOX and/or DRB were given every other day. High dose of DOX at 4 mg/kg and low dose at 1 mg/kg were used in this study. We monitored subcutaneous tumor growth and body weight of the mice during 2 weeks of the therapy. It was found that high-dose chemotherapy obviously inhibit the tumor growth, while obvious body weight loss indicated the severe toxicity of DOX (Figure 6A and 6B). In comparison with high-dose DOX chemotherapy, combine low-dose DOX and DRB successfully restrained the tumor growth as effective as high-dose chemotherapy and did not exhibit obvious adverse effect which indicated by slight body weight loss (Figure 6A and 6B). Additionally, analysis of isolated tumors from the combination treatment group showed remarkable reduction of the phosphorylated ARC expression while the total expression of ARC remains constant (Figure 6C). The reduction of phosphorylated ARC may contribute to enhanced apoptosis, as assessed by the terminal deoxynucleotidyl transferase dUTP nick end labeling (TUNEL) assay (Figure 6D) in xenograft model. These findings were consistent with our obtained results *in vitro*. In summary, our data indicate that CK2 inhibitor combined with low-dose DOX could synergistically down-regulate the phosphorylation of ARC and then induce cancer cells sensitive to undergoing apoptosis *in vivo*.

## DISCUSSION

Chemotherapy has an important role in the treatment of a variety of cancers. However, the major problem is that cancer cells are resistant to a variety of therapeutic drugs such as DOX. It is urgent to explore potential mechanisms involved in chemotherapy resistance and to get over this problem. Our current study found that cancer resistance to chemotherapy has a close relationship with the phosphorylated ARC which locates to mitochondrion in cancer cells. CK2 inhibitors could sensitize cells to apoptosis by lowering the ARC phosphorylation levels. Furthermore, we identified CK2α and CK2α' translates to nuclear in cancer cells under DOX treatment and this may go a step further to lower the ARC phosphorylation level by reducing the CK2 amount in cytoplasm. Finally, the hypothesis that ARC phosphorylation abolished by CK2 inhibitor could make cancer cell more sensitive to DOX was supported by the results in nude mice model.

DOX has been used for treating cancer for over 40 years [24] and has shown great treatment potential. It has been shown that anticancer action of DOX is elicited through DNA damage and/or reactive oxygen species generation by redox reaction. DOX can activate the apoptosis pathway by AMPK (AMP-activated protein kinase inducing apoptosis) signaling [25]. Our present study showed that DOX could make CK2α translocate from cytoplasm to nucleus meanwhile the non-phosphorylated ARC locates to cytoplasm. This means that ARC cannot be phosphorylated effectively as an active anti-apoptosis state under the DOX treatment as less catalytic CK2 available. We provide a novel explanation for DOX inducing apoptosis by down regulating ARC phosphorylation levels.

ARC as an endogenous apoptosis inhibitor can antagonize both intrinsic and extrinsic apoptosis signaling pathway [26]. Our previous works have proved that highly expressed ARC contributed to chemotherapy resistance in cancer cells and our present study revealed that ARC was phosphorylated and localized to mitochondria in HeLa and SGC-7901 cells. Our results further suggested the T149 phosphorylation of ARC was necessary to inhibit apoptosis and mitochondrial fission under DOX treatment. ARC T149 phosphorylation is required for its relocalization to mitochondria where ARC plays the anti-apoptosis function. To our knowledge, there are no publications that ARC can be phosphorylated by other kinases except for CK2. Our results suggested that p38, JNK or ERK did not phosphorylate ARC at threonine 149. Whether ARC can be phosphorylate by other kinase at other sites and whether these sites affect the localization or function of ARC need to be explored in the further study. Mitochondrial fission is involved in the initiation of apoptosis and our previous study has revealed ARC inhibits dynamin-related protein-1 (Drp1) accumulation in mitochondria and the consequent mitochondrial fission [9]. It is important to clarify how the phosphorylated ARC which localizes to mitochondrion participates in mitochondrial fission machinery. It would be interesting to understand the relationship between ARC and the mitochondrial dynamic related proteins such as Drp1, mitofusin and other factors [27].

CK2 has hundreds substrates and participates in complex regulation networks [16]. It has long been known involved in cell growth and proliferation, moreover, CK2 can suppress apoptosis [28]. Elevated CK2 activity has been associated with tumorigenesis while the precise roles it plays remain incompletely understood. CK2 can also regulate the activity of tumor suppressor proteins and oncogenes. For instance, CK2 phosphorylates p53 at Ser 392 in response to DNA damage [29]; regulates c-myc protein stability in lymphomagenesis [30]; promotes aberrant activation of nuclear factor-kappa B (NF-κB) in breast cancer cells [31]. These CK2-targeted factors have multiple functions like the regulation of cell proliferation, growth arrest, or apoptosis, the direct evidence as to whether phosphorylation eventually results in the control of apoptosis is questionable and our work showed that CK2 directly targeted ARC to inhibit apoptosis of cancer cells.

Nuclear translocation of CK2 in some cancer cells is a common downstream response of cells subjected to diverse types of growth stimuli, such as heat shock, UV irradiation and chemical-stress [32, 33]. For example, CK2 translocates to nuclear matrix with a decrease in the cytosolic fraction in human prostate carcinoma cells under androgens and/or growth factors stimuli [34]; CK2α targets to nuclear matrix in response to heat shock may serve a protective role in the cell response to stress [35]; CK2α partially localizes to perinuclear structures while a marked nuclear translocation of CK2α' occurs following ionizing radiation [36]; chemical-induced apoptosis in prostate cancer and other cells by etoposide evokes an enhancement shutting of CK2 to the nuclear matrix from the cytoplasmic [37]; CK2β subunits are retained in the cytoplasm while CK2α and CK2α' subunits are shuttled to the nucleus upon hypoxic treatment [38]. And our present work has shown that catalytic subunits CK2α and CK2α' translocate to nuclear while the regulatory subunits CK2β remain constant in cancer cells when treated with DOX. The function of CK2 locates to nuclear is very complicated, such as regulate cell cycle progression [39], response to DNA damage [40], modulate the nuclear architecture [41] and so on. Meanwhile, our results showed that the localization of ARC in mitochondrial was decreased in cancer cells treated with DOX. So we suppose that CK2 translocate to nuclear will catalyze the phosphorylation of ARC less effectively as unphosphorylated ARC predominantly distributed in cytoplasm in cancer cells. And the decrease of phosphorylated ARC level consequentially makes cancer cells sensitive to apoptosis. It would be interesting to explore the function of CK2 in nuclear under this condition and the mechanism by which CK2α transfer to nuclear in cancer cells in response to DOX remains to be determined.

CK2 activity has been found to be consistently enhanced in many human cancers. As CK2 regulates many oncogenic pathways and processes that play important roles in drug resistance, it's a promising therapeutic target for cancer therapy. As it's mentioned above that drug resistance and potential side effects limit the DOX application in cancer treatment, combining DOX with other drugs that can effectively counteract chemotherapy resistance in cancer cells may be a worthy way to treat cancer. It's reported that combination therapy with DOX even at a lower dose plus gefitinib strongly suppressed neuroblastoma growth than treatment with DOX alone [42]. There are results also demonstrate that combination of CK2 inhibitor CX-4945 with erlotinib (EGFR tyrosine kinase inhibitor) results in synergistic killing of cancer cells by attenuating the PI3K-Akt-mTOR pathway [43]. More results show that TBB treatment combined with TRAIL is a potential therapy against androgen-refractory prostate cancer [44]. In our study, we lowered the dose of DOX usage combined with DRB in xenograft models and found this combination therapy successfully restrained the tumor growth by enhancing apoptosis. Meanwhile the therapy also did not show severe toxicity indicated by slight body weight loss *in vivo*. Our results indicate that DOX at a low dose combined with CK2 inhibitors can increase therapeutic efficacy while reduce the toxicity at the same time, and this may represent a novel therapeutic concept for cancer treatment.

Taken together, we report here that phosphorylation of ARC by CK2 contributes to chemotherapy resistance by inhibiting DOX induced apoptosis, whereas, CK2 inhibitor increase the sensitivity of cancer cells to DOX by inhibiting the phosphorylation of ARC. Our results suggest that combination therapy with DOX and CK2 inhibitor might be a promising way for the treatment of cancers to increase therapeutic efficacy, reduce toxicity, and decrease the incidence of drug resistance.

## MATERIALS AND METHODS

### Reagents and cell culture

DOX, DRB and TBB were purchased from Sigma (St. Louis, MO, USA). p38 inhibitor SB203580, JNK inhibitor SP600125 and ERK inhibitor SCH772984 were purchased from were purchased from Cell Signaling Technology (Massachusetts, USA), Sigma-Aldrich (St. Louis, USA), Selleck Chemicals (Houston, USA) respectively. Anti-ARC antibody, anti-CK2α antibody, anti-CK2α' antibody and anti-CK2β antibody were obtained from Abcam (Cambridge, UK), anti-PCNA antibody was obtained from Santa Cruz Biotechnology (Texas, USA.), anti-COX IV antibody was obtained from Cell Signaling Technology (Massachusetts, USA). Anti-Phospho-T149 antibody was generated as we described [10]. Human cervical cancer cells HeLa and human gastric cancer cell line SGC-7901 were as we previously described [9]. The cells were cultured in Dulbecco's modified Eagle's medium (GIBCO, Grand Island, NY, USA) supplemented with 10% fetal bovine serum, 100 units/ml penicillin, and 100 μg/ml streptomycin in a humidified atmosphere containing 5% CO_2_ at 37°C.

### Clinical gastric cancer samples

Primary gastric cancer tissues and adjacent non-tumorous tissues from patients with gastric cancer were collected from Beijing Military General Hospital (Beijing, China). These patients were randomly selected from clinical pool of the hospital's Gastrointestinal clinic, none of the patients received chemotherapy or radiotherapy. Human tissues were collected at gastroscopy and immediately frozen in liquid nitrogen. The study was approved by ethics committee of the Beijing Military General Hospital. Informed consent was obtained from all study subjects.

### Cell viability assay and caspase-3 activity assay

Cell death was determined by Trypan Blue Exclusion. The Trypan Blue-positive and Trypan Blue-negative cells were counted. Caspase-3 activity was measured using an Apo-ONE homogeneous caspase-3/7 assay kit (Promega, Madision, WI, USA) according to the manufacturer's protocol.

### Adenovirus

Adenovirus ARC, adenovirus ARC T149A mutant and adenovirus β-galactosidase (β-gal) were as we described [9, 10]. All adenoviruses were amplified in HEK-293 cells. Adenoviral infection of cancer cells was performed as we described previously [9].

### Preparation of subcellular fractions

Cells were washed twice with PBS and the pellet was suspended in 0.2 mL of buffer A [20 mmol/L HEPES (pH 7.5), 10 mmol/L KCl, 1.5 mmol/L MgCl2, 1 mmol/L EGTA, 1 mol/L EDTA, 1 mmol/L DTT, 0.1 mmol/L PMSF, 250 mmol/L sucrose] containing a protease inhibitor cocktail. The cells were homogenized by 35 strokes in a Dounce homogenizer. The homogenates were centrifuged twice at 750 g for 5 min at 4°C to collect nuclei and debris. The supernatants were centrifuged at 10,000 g for 15 min at 4°C to collect mitochondria-enriched heavy membrane pellet (HM). The resulting supernatants were centrifuged to yield cytosolic fractions.

### Immunoblotting

Cells were lysed for 1 h at 4°C in a lysis buffer (20 mmol/L Tris pH 7.5, 2 mmol/L EDTA, 3 mmol/L EGTA, 2 mmol/L DTT, 250 mmol/L sucrose, 0.1 mmol/L phenylmethylsulfonyl fluoride, 1% Triton X-100) containing a protease inhibitor cocktail. Protein samples were subjected to 12% SDS-PAGE and transferred to nitrocellulose membranes. Blots were probed using corresponding primary antibodies. Then the horseradish peroxidase-conjugated secondary antibodies were used. Antigen-antibody complexes were tested by enhanced chemiluminescence.

### Immunoprecipitation

Cells were lysed for 1 h at 4°C in a lysis buffer. To perform immunoprecipitation, the cell lysates were precleared with 10% (vol/vol) protein A-agarose (Roche) for 1 h on a rocking platform. Specific antibodies or normal rabbit IgG as negative control were added and rocked for 1 h. Immunoprecipitates were captured with 10% (vol/vol) protein A-agarose for another hour. The agarose beads were spun down and washed thrice with lysis buffer. The antigens were released and denatured by adding SDS sample buffer.

### Mitochondrial staining and analysis of mitochondrial fission

Cells were plated onto the cover-slips coated with 0.01% poly-L-lysine. After treatment they were stained for 20 min with 0.02 μmol/L MitoTracker Red CMXRos (Molecular Probes) and the cell nuclei were stained by 600 nM 4′, 6′-diamino-2-phenylindole (DAPI) (Sigma-Aldrich) for 5 min. Immunofluorescence analysis of mitochondrial fission was performed as we described with modification [45]. The samples were imaged using a laser scanning confocal microscope (Zeiss LSM 510 META). Images were assessed in random order. A punctiform mitochondrial phenotype was scored when at least 90% of the tubular mitochondria were disintegrated.

### TUNEL analysis

TUNEL assay was performed using a kit from Roche Applied Science (Hamburg, Germany). The procedures were following the kit instructions. The samples were imaged using a laser scanning confocal microscope (Zeiss LSM 510 META).

### Subcutaneous tumor xenograft model

For the combination therapy experiments in Figure 6A, 1 × 10^7^ HeLa cells were injected subcutaneously into the right flanks of female BALB/c nude mice (4–5 weeks old). When tumors reached an average volume of 250–300 mm^3^, the mice were randomly divided into 5 groups, 6 mice in each group. According to the experimental design, intraperitoneal delivery of DOX and/or DRB was administrated every other day. Two different doses of DOX (1 mg/kg, 4 mg/kg) were used. During DOX and/or DRB treatment, the tumor size and body weight of the mice were monitored every other day. The tumor volume was calculated using the formula volume = length × width^2^/2. At the end of the experiment, the mice were sacrificed and the tumors were separated for further analysis. Animal experiments were reviewed and approved by the Animal Care Committee, Institute of Zoology, Chinese academy of Sciences.

### Statistical analysis

All statistical analyses were performed using the SPSS 13.0 statistical software package. The results are expressed as means ± SD of at least three independent experiments. The differences among experimental groups were evaluated by one-way analysis of variance. Paired data were determined by two-tailed Student's *t*-test. *p* < 0.05 was considered statistically significant.

## SUPPLEMENTARY FIGURES



## Abbreviations

ARC: apoptosis repressor with caspase recruitment domain
CK2: casein kinase II
DOX: doxorubicin
TBB: 4,5,6,7-tetrabromobenzotriazole
DRB: 5,6-dichloro-1-β-D-ribofuranosylbenzimidazole
HM: mitochondria-enriched heavy membranes
β-gal: β-galactosidase
DAPI: 4′, 6′-diamino-2-phenylindole
TUNEL: terminal deoxynucleotidyl transferase dUTP nick end labeling
