# Supplementary material for: Phosphorylation of apoptosis repressor with caspase recruitment domain by protein kinase CK2 contributes to chemotherapy resistance by inhibiting doxorubicin induced apoptosis
Source: Oncotarget. 2015 Jun 27;6(29):27700–13. doi: 10.18632/oncotarget.4392 (PMC4695019; doi:10.18632/oncotarget.4392)
Supplement: Supplementary file 1 [file oncotarget-06-27700-s001.pdf]

SUPPLEMENTARY FIGURES

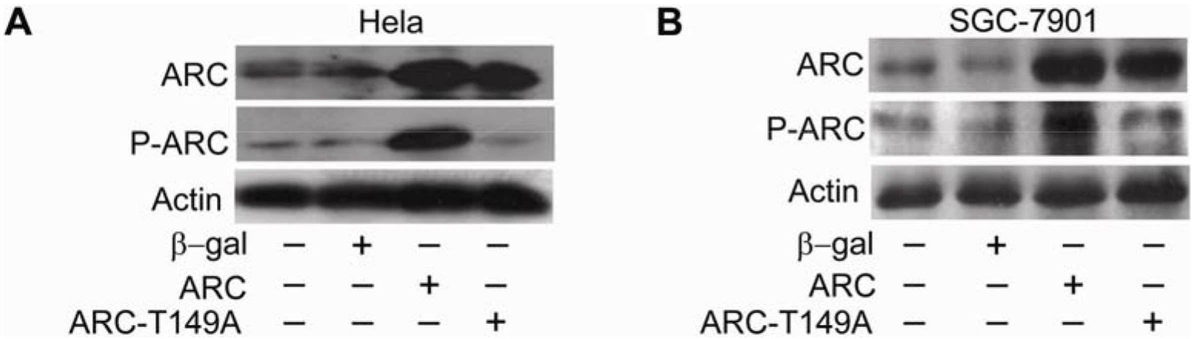

Supplementary Figure 1: Enforced expression of ARC was detected. HeLa **A.** or SGC-7901 **B.** cells were infected with adenoviral ARC or ARC T149A. Total ARC and p-ARC expression levels were analyzed by immunoblot.

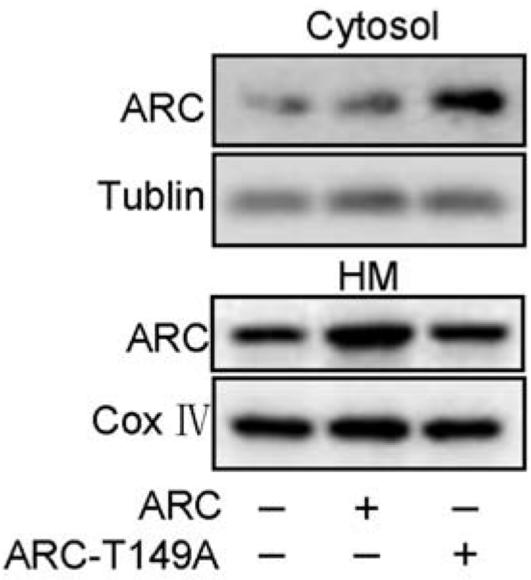

Supplementary Figure 2: Distributions of exogenous ARC and ARC T149A were detected. HeLa cells were infected with adenoviral ARC or ARC T149A and harvested for the detection of ARC in the cytosol (top) and mitochondria-enriched HM by immunoblot. A representative blot of three independent experiments is shown.

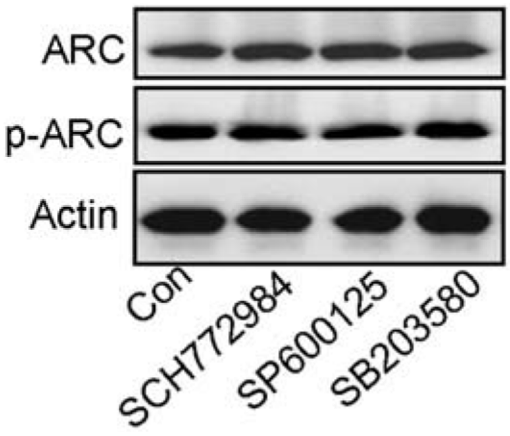

Supplementary Figure 3: The inhibitors of protein kinases ERK, JNK or p38 were not able to inhibit endogenous ARC phosphorylation. HeLa cells were treated with ERK inhibitor SCH772984, JNK inhibitor SP600125 or p38 inhibitor SB203580 for 36 hours respectively, total ARC and p-ARC were detected by immunoblot. A representative blot of three independent experiments is shown.

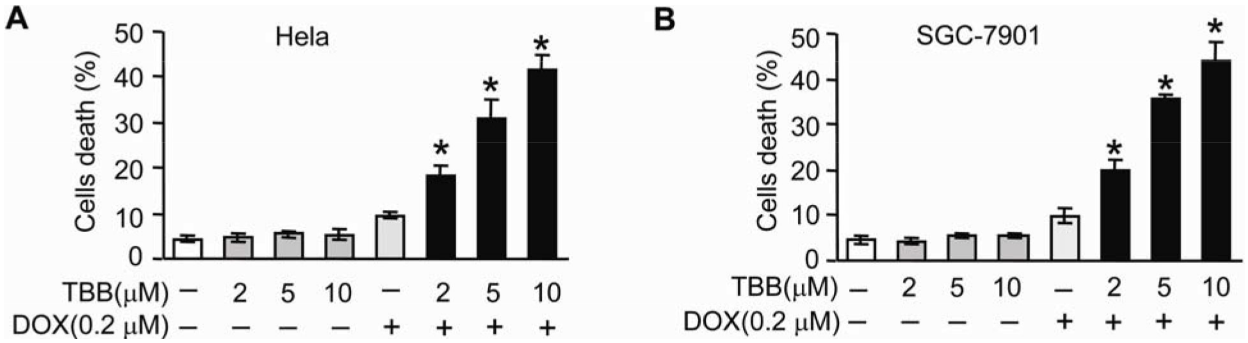

Supplementary Figure 4: TBB sensitized DOX to induce cell death. HeLa **A.** and SGC-7901 **B.** were administrated with indicated concentration of TBB for 36 hours and then treated with DOX (0.2 μM) for 36 hours. Cell death was analyzed by trypan blue exclusion. *$p < 0.05$ vs DOX alone. Data are expressed as the mean ± SD of 3 independent experiments.
